# The genetics and neuropathology of frontotemporal lobar degeneration

**DOI:** 10.1007/s00401-012-1029-x

**Published:** 2012-08-14

**Authors:** Anne Sieben, Tim Van Langenhove, Sebastiaan Engelborghs, Jean-Jacques Martin, Paul Boon, Patrick Cras, Peter-Paul De Deyn, Patrick Santens, Christine Van Broeckhoven, Marc Cruts

**Affiliations:** 1 Institute Born-Bunge, University of Antwerp, Antwerpen, Belgium; 2 Neurodegenerative Brain Diseases Group, VIB Department of Molecular Genetics, University of Antwerp, CDE, Universiteitsplein 1, 2610 Antwerpen, Belgium; 3 Department of Neurology, University Hospital Ghent and University of Ghent, Ghent, Belgium; 4 Department of Neurology, University Hospital Antwerp, Antwerpen, Belgium; 5 Department of Neurology and Memory Clinic, Hospital Network Antwerp Middelheim and Hoge Beuken, Antwerpen, Belgium; 6 Alzheimer Research Center, University Medical Center Groningen, Groningen, The Netherlands

**Keywords:** Frontotemporal lobar degeneration, Proteinopathy, MAPT, GRN, C9orf72, VCP, CHMP2B, Tau, TDP-43, FUS

## Abstract

Frontotemporal lobar degeneration (FTLD) is a heterogeneous group of disorders characterized by disturbances of behavior and personality and different types of language impairment with or without concomitant features of motor neuron disease or parkinsonism. FTLD is characterized by atrophy of the frontal and anterior temporal brain lobes. Detailed neuropathological studies have elicited proteinopathies defined by inclusions of hyperphosphorylated microtubule-associated protein tau, TAR DNA-binding protein TDP-43, fused-in-sarcoma or yet unidentified proteins in affected brain regions. Rather than the type of proteinopathy, the site of neurodegeneration correlates relatively well with the clinical presentation of FTLD. Molecular genetic studies identified five disease genes, of which the gene encoding the tau protein (*MAPT*), the growth factor precursor gene granulin (*GRN*), and *C9orf72* with unknown function are most frequently mutated. Rare mutations were also identified in the genes encoding valosin-containing protein (*VCP*) and charged multivesicular body protein 2B (*CHMP2B*). These genes are good markers to distinguish underlying neuropathological phenotypes. Due to the complex landscape of FTLD diseases, combined characterization of clinical, imaging, biological and genetic biomarkers is essential to establish a detailed diagnosis. Although major progress has been made in FTLD research in recent years, further studies are needed to completely map out and correlate the clinical, pathological and genetic entities, and to understand the underlying disease mechanisms. In this review, we summarize the current state of the rapidly progressing field of genetic, neuropathological and clinical research of this intriguing condition.

## Introduction

### History and significance

Frontotemporal lobar degeneration (FTLD) is an anatomopathological descriptive term referring to a disorder characterized by the relatively selective atrophy of the frontal and anterior temporal lobes of the brain. Apart from this commonality, FTLD is a clinically, genetically and pathologically heterogeneous group of disorders. Because disease onset occurs before the age of 65 years in 75–80 % of the patients, FTLD is considered a presenile dementia [110, 122]. In the age group between 45 and 65 years, the prevalence of FTLD has been estimated between 10 and 30 per 100,000 [11, 136], but large-scale epidemiological studies are lacking. FTLD is the second most common form of early-onset dementia, after Alzheimer’s disease (AD) [19].

FTLD has a markedly strong familial component: 30–50 % of the FTLD patients report at least one relative with similar symptomatology, and in 10–23 %, the disease segregates in the family with an autosomal dominant inheritance pattern [56, 57]. Five disease genes have been identified to cause FTLD. Further, FTLD is associated with genes that typically cause a clinical picture of amyotrophic lateral sclerosis (ALS), the most common type of motor neuron disease (MND). Common genetic, clinical and pathological characteristics between FTLD and ALS are manifold. In this era of rapidly evolving insights into the pathophysiology of this challenging group of diseases, the present review aims to provide a timely picture of the clinical, neuropathological and genetic findings in FTLD and the correlations between them.

### Clinical phenotypes of FTLD and related conditions

FTLD can manifest as two clinically recognized subtypes based on the presenting and predominant features of either behavioral and personality changes, or language disturbances. The behavioral variant of frontotemporal dementia (bvFTD) is characterized by severe changes in behavior and personality such as disinhibition, apathy, loss of empathy, or stereotypic behavior, leading to a loss of social competence [73, 109, 127, 151]. Executive functions are impaired, while at least in the initial stages of the disease, memory and perceptuospatial skills are well preserved. As the differential diagnosis in patients with psychiatric disturbances or AD is not always straightforward, the ‘International Behavioral Variant FTD Criteria Consortium’ developed international consensus criteria for bvFTD. According to these criteria, subclassifications were made in possible bvFTD defined by clinical criteria, probable bvFTD supported by neuro-imaging data, and definite bvFTD confirmation by neuropathological evidence or a pathogenic mutation [127]. bvFTD accounts for more than 50 % of the FTLD patients. The onset of bvFTD is typically before the age of 65 years, with an average onset age of 58 years [71, 73].

If the patient presents with language difficulties, a diagnosis of primary progressive aphasia (PPA) is made. PPA was originally further categorized into progressive nonfluent aphasia (PNFA) and semantic dementia (SD) [73]. However, the clinical picture of a number of PPA patients did not fit either diagnosis, which led to the description of the third variant, logopenic progressive aphasia (LPA). The lack of clear definitions of the three subtypes led in 2011 to new recommendations for the subclassification of PPA into nonfluent/agrammatic variant PPA (the former PNFA), semantic variant PPA (the former SD) and the logopenic variant PPA (also known as LPA) [59]. Nonfluent/agrammatic variant PPA or PNFA is characterized by effortful speech and grammatical error-making, with relatively preserved language comprehension. Apraxia of speech (AOS) or orofacial apraxia is frequently accompanying the aphasia [73]. PNFA is the second most prevalent presentation of FTLD, accounting for a large 25 % [71]. Semantic variant PPA or SD presents with impaired comprehension and conceptual knowledge with concomitant development of anomia, while speech production is spared [59, 109, 151]. SD presents in 20–25 % of the FTLD patients [71]. LPA is mostly associated with a neuropathological diagnosis of AD [121] and is not considered part of the FTLD group of disorders. Based on the evidence supporting the diagnosis of PPA, the label “possible” (clinical features), “probable” (clinical findings in combination with neuro-imaging) and “definite” (after post-mortem examination or when a gene mutation is known) are provided [59, 125].

Overlap between the clinical syndromes of bvFTD, PNFA and SD can occur during the progression of the disease and clinical distinction between them is often complicated in advanced disease stages. The median survival from the onset of symptoms is 6–11 years, independent of age at onset or gender [63, 130]. While some studies show no differences in survival rates among the clinical subtypes of FTLD, other studies suggested shorter survival in bvFTD and longest survival in SD [63, 71, 115, 130]. The gender distribution of FTLD appears to vary by the clinical syndrome, with a male preponderance in the behavioral and personality disorders and a female predominance in the language disorders [71].

The comorbidity of ALS with behavior alterations, cognitive impairment or dementia has been noticed since the early 20th century [91, 119, 129]. FTLD may precede, follow or coincide with the onset of motor symptoms [172]. In one of the largest studies on neuropsychological disturbances in ALS, 47 % of the patients showed some degree of dysfunction in frontal lobe tests, which was in 15 % sufficient for a diagnosis of FTLD [129]. Conversely, motor neuron dysfunction has been described in 40 % of the FTLD patients, and the diagnostic criteria for ALS were met in 15 %, referred to as FTLD-ALS [18, 90]. FTLD-ALS patients have a poor prognosis with a mean survival of 2–3 years from the onset of first symptoms [63, 73]. The reported heritability of FTLD-ALS is high: approximately 50 % is considered familial [56, 132, 141].

Apart from MND, other conditions are closely related to FTLD, including progressive supranuclear palsy (PSP) syndromes, corticobasal syndrome (CBS), FTD with parkinsonism (FTDP) and argyrophilic grain disease (AGD). The most common clinical syndromes are PSP syndromes with a prevalence of 3.1 per 100,000, followed by CBS with a prevalence of less than 1 per 100,000 [41]. PSP was originally described as a clinical syndrome characterized by extrapyramidal symptoms and a progressive dementia. Patients present with a symmetrical, akinetic rigid parkinsonism, severe postural instability and supranuclear ophthalmoplegia. Most patients develop the disease at middle or late age (75 ± 8 years) and death occurs on average 7 years later [35]. In more recent years, the spectrum of PSP syndromes has been extended to not only include classical PSP syndrome, termed the Richardson’s type, but also PSP-parkinsonism, which presents as a more asymmetrical disorder resembling Parkinson’s disease [177] and the syndrome of pure akinesia characterized by freezing of gait and speech dysfluencies as most prominent features [34, 177]. CBS most frequently presents as a combined clinical picture, consisting of a focal cortical deficit (e.g. limb apraxia, aphasia, frontal lobe syndrome, alien limb phenomenon, cortical sensory loss) and a progressive asymmetrical movement disorder (myoclonus, dystonia, tremor). In a later stage of the disease, patients often develop cognitive dysfunctions, sometimes in combination with a frontotemporal behavioral syndrome. The term FTDP-17 was defined in 1997 [47, 153], describing 13 families presenting with a clinical syndrome of autosomal dominant disinhibition, dementia, parkinsonism, and amyotrophy and showing genetic linkage to chromosome 17. Nowadays, we are able to differentiate mutations in the *MAPT* or to the *GRN* gene, both located on chromosome 17. The clinical picture resembles bvFTD, while cognitive deficits include anterograde memory dysfunction in an initial stage, later accompanied by progressive deterioration of visuospatial function, orientation and global memory. Eventually, mutism occurs. Motor signs typically include the development of symmetrical bradykinesia without resting tremor, in combination with axial rigidity and postural instability. There is poor or no effect of levodopa therapy. Other motor symptoms include vertical gaze palsy, dystonia, upper and lower motor dysfunction, eye lid apraxia and dysphagia [35]. The onset age can range from the early 20s to the late 70s, with an average of 50 years. The clinical early presentation of AGD is similar to AD but disease progression is less aggressive, with patients having a clinical picture resembling mild cognitive impairment (MCI) for many years [35, 44]. AGD accounts for approximately 5 % of the neurodegenerative dementia cases, with an increasing prevalence with advancing age [35, 44]. As FTLD is a distinctive clinical, genetic and neuropathological entity, it should be noted that an FTD phenotype can also be caused by underlying AD pathology (i.e. frontal variant AD). Similarly, a clinical phenotype of CBS can be caused by AD pathology [61].

### Neuropathology of FTLD and cliniconeuropathological correlations

The FTLD brain is by definition characterized by diverse patterns of atrophy of frontal and anterior temporal lobes. Different patterns of atrophy have been described and a strong correlation with clinical phenotypes was found. A relatively symmetrical atrophy of the frontal lobes, insula, anterior cingulate and anterior temporal lobes is associated with bvFTD. An asymmetric atrophy of the left (linguistic dominant) anterior inferior temporal lobe gives rise to SD. Patients with an asymmetrical atrophy of the right anterior temporal lobe (right-sided SD) present with a behavioral syndrome similar to bvFTD. These patients develop emotional bluntness and, in some cases, loss of interest and bizarre affect. As the atrophy progresses, prosopagnosia and associative agnosia are often seen in combination with eating disorders and rigid, compulsive behavior. In patients with PNFA, an asymmetric atrophy involving the anterior perisylvian cortex, mainly of the dominant hemisphere, is seen [59, 122].

On microscopic examination, the neuronal loss and astrocytosis are seen in cortices of atrophied frontal and temporal lobes. In addition, FTLD is a proteinopathy characterized by the presence of abnormal, ubiquitinated protein inclusions in cytoplasm or nuclei of neuronal and glial cells. Adjunctive immunohistochemistry allows subcategorization of these disorders into specific proteinopathies based on the major constituent of the inclusions. From a historic perspective, two pathological categories of FTLD were initially made [20, 95, 103]: in a first group of patients, the disease presented neurons and glial cells containing inclusions of hyperphosphorylated tau protein, therefore referred to as FTLD-tau [97], including Pick’s disease (PiD). However, more than 50 % of the FTLD patients presented with tau-negative ubiquitin staining inclusions at the time of unknown composition, therefore referred to as FTLD-Ubiquitin or FTLD-U [90, 95]. In 80–95 % [94, 131] of this group, inclusions were later found to be composed of transactive response (TAR) DNA-binding protein 43 (TDP-43) [111], thus referred to as FTLD-TDP [95, 112]. A considerable number of TDP-43-negative FTLD-U cases had inclusions of fused-in-sarcoma protein (FUS), thus referred to as FTLD-FUS [96]. However, in a small number of FTLD-U patients, the inclusion protein remains unknown until today. This group is referred to as FTLD-ubiquitin proteasome system (FTLD-UPS) [36, 95].

**Fig. 1:**
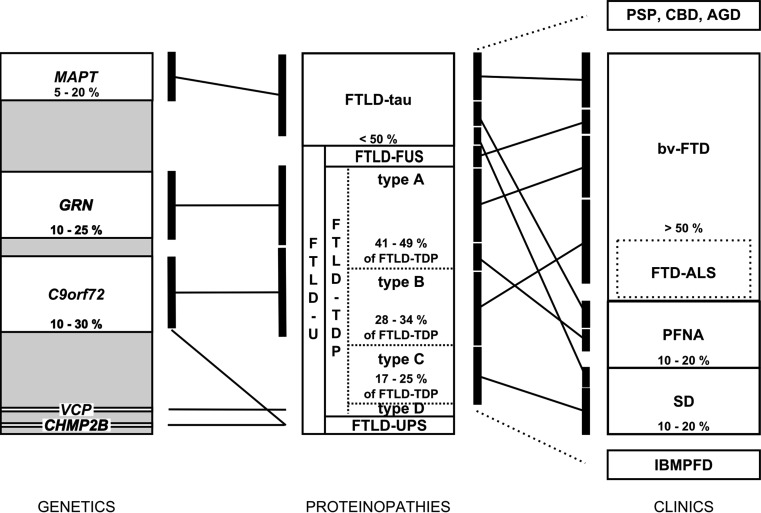
Diagram illustrating the clinical, genetic and neuropathological correlations in FTLD. The *gray background* of the genetics box represents the genetically unexplained fraction in FTLD cases overall (as compared to familial cases in Table 2)

Correlations were noted between the clinical FTLD subtypes and underlying proteinopathies, but a strict one-to-one relationship is lacking. An FTLD-FUS proteinopathy is invariantly associated with a clinical diagnosis of bvFTD, either with or without the signs of MND. However, bvFTD is also associated with FTLD-tau and FTLD-TDP (Fig. 1). Although PNFA and SD are both associated with FTLD-tau and FTLD-TDP, PNFA is commonly associated with tau pathology, especially when AOS or orofacial apraxia is present. On the other hand, SD is, in most cases, linked with TDP-43-immunoreactive pathology, although tau pathology is sometimes observed. When SD is associated with FTLD-tau, patients often present with acalculia [122]. In right-sided SD, the underlying neuropathology is mostly a TDP-43 proteinopathy. Tau pathology was also associated with FTD with parkinsonism, PSP syndromes, CBS and AGD. Similarly, TDP-43 and FUS proteinopathies are also commonly found in MND with or without FTLD.

### Molecular genetics

Positive family history is observed in 40–50 % of the FTLD patients [85] and at least 10–50 % of the patients are associated with an inheritance pattern compatible with a highly penetrant Mendelian mutation [32, 107]. When considering clinical FTLD subtypes, family history is most prominent in bvFTD (45 %), especially when concomitant symptoms of MND are present (60 %), while SD appeared to be the least hereditary FTLD subtype (<20 %) [56]. Molecular genetic studies have identified five genes that, when mutated, cause FTLD: *C9orf72*, granulin (*GRN*), the microtubule associated protein tau gene (*MAPT*), the gene encoding valosin-containing protein (*VCP*) and the charged multivesicular body protein 2B (*CHMP2B*) [30]. Mutations in common ALS genes *TARDBP* and *FUS* (see ALS review in this issue), in rare cases, present clinically with FTLD [8, 171], even without the observed signs of MND (e.g. [68]). The relative mutation frequencies of these genes vary substantially among different populations due to founder effects resulting in a regionally high occurrence of one or a limited number of specific mutations. Also, reported mutation frequencies vary significantly between studies, depending on the method of patient ascertainment (e.g. population-based vs. hospital-based) and employed inclusion criteria (e.g. clinical vs. pathological diagnosis, familial vs. non-familial or both). However, in general terms, mutations in *C9orf72*, *GRN* and *MAPT* are the most common and together explain at least 17 % of the familial FTLD (Table 2). In two genetically fully documented FTLD series, summed *C9orf72*, *GRN* and *MAPT* mutation frequencies were 32 [33] and 40 % [55]. Mutations in *VCP* and *CHMP2B* are rare, each explaining less than 1 % of the familial FTLD.

As the mutant gene is initiating the biological disease processes underlying the neuropathological changes, a relatively strict correlation between the affected gene and associated neuropathology is observed (Fig. 1). However, due to the lack of a tight correlation between the type of pathology and the clinical manifestation thereof, the correlation between the disease gene and associated clinical phenotype is limited.

In addition to the highly penetrant Mendelian FTLD genes, few susceptibility genes are reported. From the Mendelian genes, *MAPT* appears to also harbor a genetic risk to develop tauopathies [6, 64]. The only systematic genome-wide association study in FTLD reported until today identified *TMEM106B* at chromosome 7p21 as a risk factor for FTLD-TDP [165].

## Autosomal dominant genes

### MAPT

The first significant genetic linkage found in FTLD families was with markers at chromosome 17q21 [69, 120, 154]. Linkage was described in 13 families in which a consensus clinical syndrome of autosomal dominant disinhibition, dementia, parkinsonism, and amyotrophy was found, termed FTDP-17 [47]. As tau was implicated in FTLD pathology and the chromosomal location of *MAPT* coincided with the linkage at chromosome 17, *MAPT* was the most obvious candidate gene and mutations were identified in FTDP-17 families [69]. Today, 44 different *MAPT* mutations are reported in 134 FTLD families [30] (Table 2). The mutations are mainly clustered in the five most 3′ exons from 9 to 13, encoding the four microtubule-binding domains of tau. In normal brain, the tau protein occurs as six isoforms of which three contain three microtubule-binding domains (3R tau) and three contain four microtubule-binding domains (4R tau) [41]. A substantial number of mutations were located in the intron 10 splice donor site or intronic splice regulatory elements resulting in aberrant splicing of exon 10. Interestingly, *MAPT* exon 10 encodes one of the microtubule-binding domains and the mutations affecting the splicing of this exon result in aberrant ratios of 3R and 4R tau [38]. Synonymous and nonsynonymous mutations in exon 10 were shown to locate in exonic splice regulatory elements resulting in similar aberrant splicing effects [39]. In addition, missense mutations are identified that affect the amino acid sequence of the microtubule-binding domains. Mutations affect the binding of tau to tubulin either due to an increased expression of 4R tau relative to 3R tau isoforms, or due to the altered binding properties of mutant tau protein [123]. The mutations thus disturb the subtle equilibrium between cytoskeletal assembly and disassembly affecting neuronal plasticity and axonal transport across the microtubules. In addition, coding *MAPT* mutations increase the tendency of tau to form neurotoxic aggregates. Mutations in exon 10 lead to 4R tau in both neurons and glia, while mutations outside exon 10 lead to neuronal accumulation of 3R tau and 4R tau [133]. Except for p.P301L and IVS10+16C>T that occur in 32 and 27 families, respectively, *MAPT* mutations are rare and seen in single families.

**Fig. 2:**
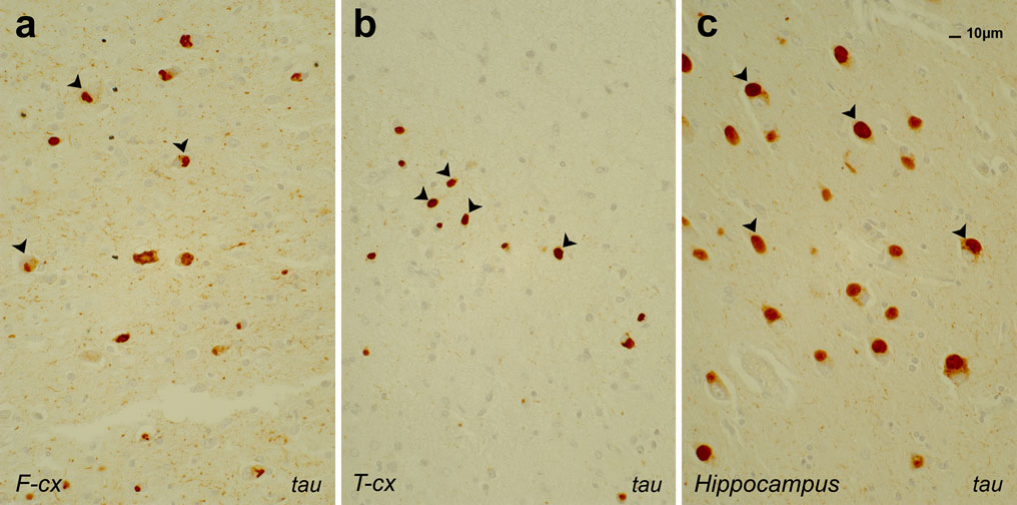
FTLD-tau pathology in brain sections of a Pick disease patient with bvFTD after immunostaining with AT8 antibody. **a** Frontal cortex (*F-cx*), **b** temporal neocortex (*T-cx*), **c** hippocampus. *Arrowheads* indicate the characteristic Pick bodies representing neuronal intracytoplasmatic spheroid tau inclusions

FTLD due to *MAPT* mutations is invariantly of the pathological type FTLD-tau, including PiD. In PiD pathology, the cardinal microscopic features are neuronal intracytoplasmatic spheroid tau-containing Pick bodies in the granular neurons of the dentate gyrus and in pyramidal neurons of the hippocampus and affected neocortical regions (Fig. 2a–c). Pick bodies, mainly localized in cortical layers II and III, and to a lesser extent also in deeper layers [41], specifically accumulate insoluble 3R tau [35, 41, 48, 73]. In addition to FTLD-tau, *MAPT* mutations have also been associated with other tauopathies including PSP, CBS, FTD with parkinsonism and the rarely seen AGD [73, 79]. The typical tau lesions in PSP are globose neurofibrillary tangles (NFT) found in neurons of the subcortical nuclei [35, 41, 73]. In CBS patients, heterogeneous tau-immunoreactive inclusions are also found in cortical neurons and neurons in substantia nigra and locus coeruleus. Other characteristic hallmarks are astrocytic plaques containing hyperphosphorylated tau and oligodendroglial inclusions [35, 41]. The neuropathological hallmark of FTD with parkinsonism is the combination of tau-immunoreactive aggregates in neurons, astrocytes and oligodendroglial cells, throughout cortex, deep gray and subcortical white matter [35, 41]. Often, these lesions are already extensive in early and intermediate stages of the disease. In AGD, on external macroscopic examination, mild diffuse cortical atrophy is seen, with a predilection of the ambient gyrus [35, 44]. The histopathological hallmark is the presence of argyrophilic grains (ArG). These ArG are present throughout the neuropil of cortical and subcortical structures. Lesions are initially present in the amygdala and temporal allocortex, spreading toward temporal neocortex as disease progresses.

FTLD-tau is mainly associated with bvFTD, however, forms of PPA are also reported [105]. Symptoms of MND are rare. On average, FTLD-tau is characterized by the earliest onset age in the FTLD syndromes, although the range of onset ages is large and strongly overlapping with other types of FTLD. A literature survey of 228 FTLD patients carrying a *MAPT* mutation demonstrated that the average onset age was 48 ± 10 years with a disease duration of 9 ± 5.5 years [27].

### GRN

In some families linked to chromosome 17q21, extensive mutation analyses could not identify a *MAPT* mutation. It became apparent that, in FTLD families negative for *MAPT* mutations, the characteristic pathological inclusions were of the tau-negative, ubiquitin-positive types suggesting that mutations in another gene at the same genetic locus were causing this FTLD-U type of disease. Extensive mutation analyses of other positional candidate genes identified mutation in the nearby *GRN* gene [7, 28]. To date, 69 different *GRN* mutations have been reported in 231 families [30] (Table 2). *GRN* mutations are distributed across the complete coding region and splice sites of the gene. They are loss-of-function mutations leading to reduced functional protein and resulting in haplo-insufficiency [52]. Most mutations produce null alleles as a result of mRNA decay mediated by a nonsense or frameshift mutation [7, 28, 50], but other mechanisms including gene deletion [53] and defective protein sorting [143] are described as well. *GRN* encodes progranulin, a ubiquitously expressed growth factor precursor consisting of 7.5 granulin peptides [1]. Both full-length progranulin and the granulin peptides are implicated in a wide range of biological processes such as inflammation and wound repair, as well as in pathological conditions including tumorigenesis [62]. Although their roles in the CNS are not well established, in vitro and in vivo studies suggest a neurotrophic function involved in neuronal survival and neurite outgrowth [2, 138, 164, 178].

The effect of *GRN* missense mutations on reduced function and resulting phenotype is not straightforward: some compromise protein stability or cellular sorting, which might result in complete loss of function and lead to haplo-insufficiency, thereby behaving as highly penetrant FTLD mutations. Clear examples of loss-of-function (LOF) missense mutations are those disrupting the signal peptide [104, 143] or affecting the disulfide bonds controlling the characteristic granulin fold [166]. Other missense mutations might only partially compromise function and behave as risk alleles [29]. *GRN* missense mutations have been implicated in AD risk [15, 150] and survival in ALS [148]. A useful measure of the reduced GRN function, discriminating between pathogenic and neutral missense mutations, is the granulin protein level in cerebrospinal fluid [22, 164], plasma [21, 45] and serum [149], although the correlation between protein expression level and associated disease risk is poorly studied.

**Fig. 3:**
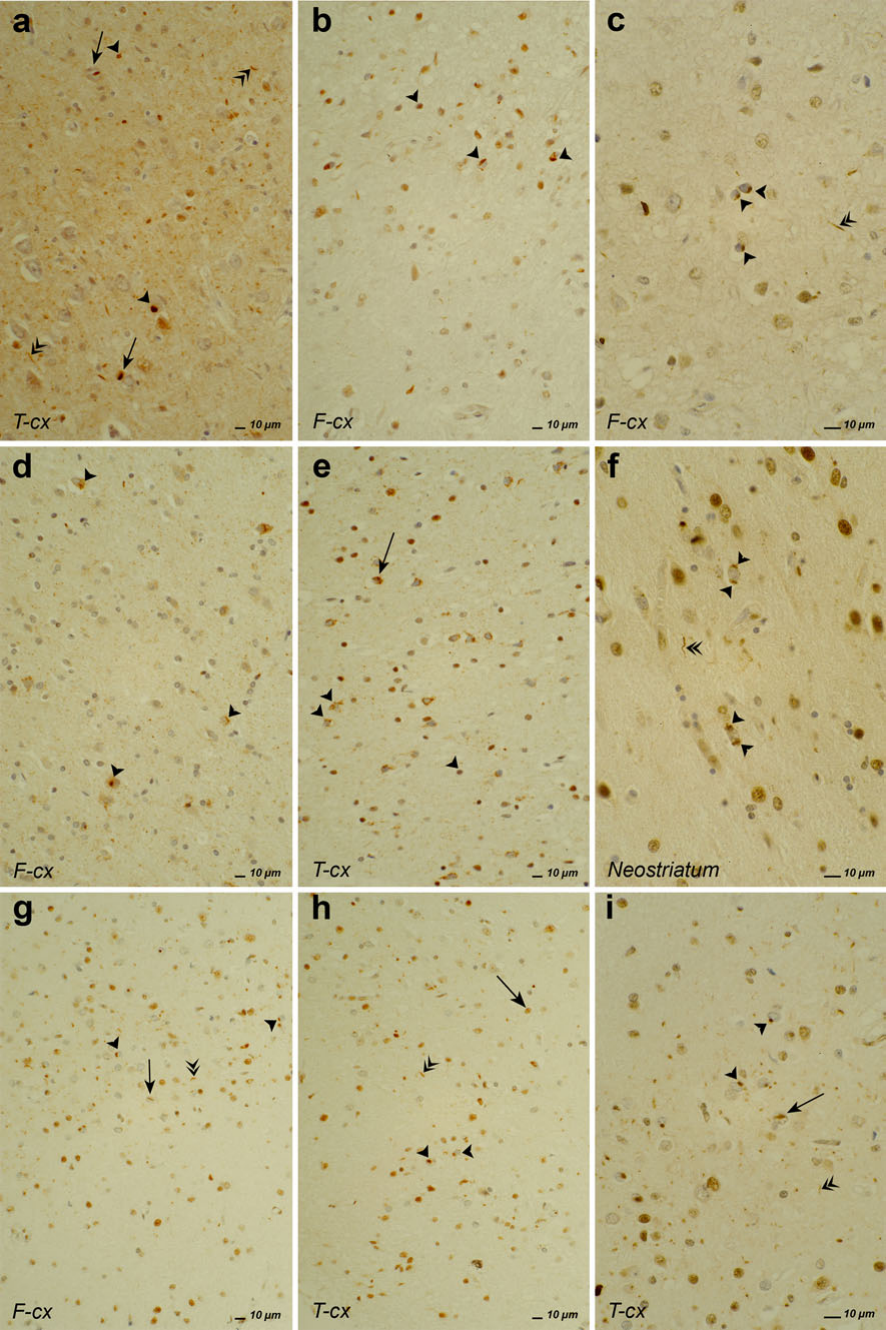
Different types of TDP-43 pathology in brains of patients with GRN mutation, C9orf72 hexanucleotide repeat expansion and VCP mutation after immunostaining with TDP-43 antibody. **a–c** TDP-43 type A pathology in a bvFTD patient of family DR8, carrying the GRN IVS0 + 5G>C mutation [14, 28]. *Arrowhead*, *double arrowhead* and *arrow* show moderate NCI, DN and NII load, respectively, in layer II of temporal (**a**) and frontal (**b**) neocortex. **c** Higher magnification of NCI load in frontal cortex. **d–f** TDP-43 type B pathology in a bvFTD patient with the C9orf72 hexanucleotide repeat expansion [55]. *Arrowhead* and *arrow* show NCI and DN, respectively, spread throughout the entire cortical thickness in frontal cortex (**d**), temporal neocortex (**e**) and NCI in neostriatum (**f**, higher magnification). **g–i** TDP-43 type D pathology in a bvFTD patient carrying the VCP p.Arg159His mutation [168]. Note the extensive NCI (*arrowhead*), NII (*double arrowhead*) and DN (*arrow*) in frontal cortex (**g**), temporal neocortex (**h, i**, higher magnification)

All *GRN*-associated FTLD patients have a FTLD-TDP proteinopathy [4, 117]. The presence of TDP-43 positive inclusions is invariably associated with a decreased physiologically normal nuclear staining [36, 95]. Recent neuropathological studies elicited four FTLD-TDP subtypes A to D based on the cortical distribution, intracellular location and morphology of the inclusions [99] (Table 1; Fig. 3). Based on the large clinicopathological studies, the most common FTLD-TDP subtype is type A accounting for 41–49 % of the cases, followed by type B with 28–34 % and type C with 17–25 %, while type D is rare [19, 73]. All *GRN* mutations are associated with FTLD-TDP type A (Fig. 3a–c), and in turn mutations in *GRN* explain about 40 % of these cases [133]. In FTLD-TDP type A, many neuronal cytoplasmic inclusions (NCI), short dystrophic neurites (DN) and some lentiform neuronal intranuclear inclusions (NII) are observed in layer II of the affected neocortex. NCI in the dentate granular gyrus of the hippocampus are variable in number [99].

**Table 1:** Major genetic causes, clinical phenotypes and neuropathological characteristics of FTLD-TDP subtypes

Subtypes of FTLD-TDP	Associated gene mutations^a^	Associated clinical phenotype	Neuropathological findings
Type A	*GRN, C9orf72*	bvFTD (PNFA)	Many NCI and DN in superficial cortical layers (layer II) NII in superficial cortical layers GCI
Type B	*C9orf72*	FTD-MND bvFTD	NCI throughout the entire cortical thickness NCI in hypoglossal nucleus and in ventral horn of the spinal cord Pre-inclusions
Type C		SD (bvFTD)	Long DN in superficial cortical layers Few NCI and NII
Type D	*VCP*	IBMPFD	Many NII and DN throughout the entire cortical thickness Few NCI

*bvFTD* behavioral variant frontotemporal dementia, *PNFA* primary nonfluent aphasia, *FTD-MND* frontotemporal dementia with motor neuron disease, *SD* semantic dementia, *IBMPFD* inclusion body myopathy, Paget disease of bone and frontotemporal dementia, *NCI* neuronal cytoplasmic inclusions, *NII* neuronal intranuclear inclusions, *GCI* glial cytoplasmic inclusions, *DN* dystrophic neurites

^a^See Table 2 for full gene names

Despite the fact that haplo-insufficiency is the common disease mechanism in all patients carrying a *GRN* mutation, the associated clinical phenotype is variable, including bvFTD and PNFA [88, 125]. Parkinsonian symptoms are often observed, but motor neuron symptoms are rare [23]. Sporadically, clinical diagnoses of related neurodegenerative diseases including AD and parkinsonian disorders have been associated with *GRN* mutations [14, 21, 22, 88]. A literature survey of 183 patients with *GRN*-associated FTLD demonstrated that the onset age was on average 61 ± 9 years and 13 years later than in *MAPT* mutation carriers although as in FTLD-tau, onset ages are highly variable, ranging from 35 to 87 years [29, 30]. A considerable age-dependent penetrance was described with 50–60 % of the mutation carriers being affected by the age of 60 years and 90–95 % by the age of 70 years [50, 125]. Consequently, family history is not always apparent and *GRN* mutations are found in a significant number of FTLD patients classified as being sporadic [87].

### C9orf72

Genetic linkage [12, 54, 89, 93, 102, 162, 163] and association [83, 144, 170] studies in FTLD-TDP, FTLD-ALS and ALS suggested a common genetic defect located at chromosome 9p21 in the complete spectrum of diseases in the FTLD–MND complex [55]. Extensive mutation analyses of positional candidate genes identified an expanded noncoding G_4_C_2_ hexanucleotide repeat in *C9orf72* explaining linkage and association [33, 55, 128]. In the normal population, the size of the G_4_C_2_ repeat ranges from 3 to 25 units, which is expanded to at least 60 units in patients [33, 55, 128]. Accurate size estimations of the expanded repeat in a substantial number of carriers are lacking, as the commonly used detection method based on the repeat-primed PCR does not differentiate repeat sizes >60 units. In one study, Southern blot analyses of a limited number of repeat expansion carriers estimated repeat sizes between 700 and 1,600 units [33]. In an extended study, pathological *C9orf72* repeat expansions were observed in 11.4 % of the 1,381 FTLD patients of European origin, rising to 24.8 % in familial patients [101]. The same study reported a *C9orf72* repeat expansion frequency of 6.0 % in sporadic FTLD. Whether this high mutation frequency in patients without family history of disease is explained by a high de novo repeat expansion rate remains to be determined, but a preliminary study suggests that this is more likely explained by incomplete penetrance [116].

*C9orf72* encodes a ubiquitously expressed protein of unknown function. It is expressed as three major transcripts [33] and the expanded G_4_C_2_ repeat is located in the proximal regulatory region of *C9orf72* [55], upstream of one and in the first intron of the two other transcripts. Repeat expansion results in near complete loss of expression of the major gene transcripts [33, 55, 128]. In addition, accumulation of transcripts harboring the expanded G_4_C_2_ repeat in nuclear RNA foci was described [33] although this was not replicated in another study [146]. Whether haplo-insufficiency due to the loss of transcription, RNA toxicity due to sequestration of RNA-binding proteins in RNA foci, and/or yet unidentified mechanisms are contributing to disease, needs further investigation.

Before the identification of the *C9orf72* repeat expansions, FTLD linked to chromosome 9p21 was neuropathologically characterized as being of the TDP-43 proteinopathy type B. The pathological characteristics of FTLD-TDP type B (Fig. 3d–f) include NCI throughout the entire cortical thickness [99]. NCI are seen in the granular cells of the dentate gyrus of the hippocampus. In some patients, NCI can also be found in the motor neurons of the hypoglossal nuclei and ventral horn of the spinal cord. Next to these TDP-43 inclusions, granular “pre”-inclusions are seen in affected cortical regions [36]. Ubiquitin staining of these pre-inclusions is negative. Furthermore, GCI are often seen in brain stem and spinal cord [36]. When the pathological *C9orf72* repeat expansion was identified, a larger number patients with neuropathological documentation could be attributed to this mutation, revealing a wider pathological diversity. In a substantial number of patients, the number and distribution of TDP-43 inclusions were more consistent with a TDP-43 type A proteinopathy [107] or intermediate to type B and type A proteinopathy [152]. Alternatively, the TDP-43 lesion load could be relatively limited or even absent resulting in a pathological diagnosis of FTLD-UPS in rare patients [55, 107]. Abundant p62 positive, TDP-43 negative NCI and rare NII were seen in the pyramidal cell layer of the hippocampus and cerebellar granular layer of most patients [3, 13]. Remarkably, similar brain inclusions were also observed in ALS patients without associated cognitive decline [158]. Interestingly, the sequestosome 1 gene (*SQSTM1*) encoding the p62 protein harbors mutations leading to ALS [42] and Paget disease of bone (PDB) [58]. Nevertheless, p62 immunoreactivity of the TDP-43-negative NCI might indicate general staining of proteins of the ubiquitin–proteasome system (UPS) for protein degradation, and the hallmark protein of these aggregations remains to be identified. The presence of DN and aggregates that were immunoreactive to ubiquilin (UBQLN) in the molecular layer of the hippocampus and throughout the neocortex was shown to be highly specific for the *C9orf72* mutation. Further, UBQLN and p62 colocalized in NCI in the dentate gyrus of the hippocampus and the granular layer of the cerebellum [13]. Staining of C9orf72 itself has until today remained inconclusive. Polyclonal antibodies against both protein isoforms showed a punctiform staining of synaptic terminals in the CA4 region of the hippocampus in patients with the C9orf72 mutation, but also in patients with AD and controls [13, 33, 146].

*C9orf72* repeat expansions clinically present with a widely variable phenotype including FTLD, ALS or FTLD-ALS [33, 55, 128]. With a mutation frequency of 30 % in FTLD-ALS [55], it is the only known common disease gene in this condition. Independent of concomitant ALS, FTLD is mostly of the behavioral type but patients with PPA have been described [33, 55, 146, 152]. Reported mean onset ages ranged between 55.3 and 58.3 years [33, 55, 146, 152] and are intermediate to onset ages in FTLD associated with *MAPT* and *GRN*. Also here, a high variability in onset age was noted, even among patients of the same family [33, 55]. Typical of repeat expansion diseases, genetic anticipation has been suggested with decreasing onset ages in younger generations [5, 24, 43, 156], and a decrease in onset age of 7 years in the younger of two subsequent generations has been suggested for ALS [24]. However, reports correlating sizes of the expanded repeat allele and onset age are lacking.

### VCP

*VCP* mutations were identified by linkage analysis studies in autosomal dominant families with disabling muscle weakness due to inclusion body myopathy (IBM), osteolytic bone lesions consistent with PDB and FTLD (IBMPFD) [174]. Today, 17 different mutations have been identified in 41 independent families [30]. Incomplete penetrance was noted for all three clinical characteristic features and patients may present with classical FTLD.

*VCP* encodes a ubiquitously expressed member of a family of ATPases associated with a wide range of cellular functions through interactions with different adaptor proteins [175]. All IBMPFD mutations reside at the interface between the D1 ATPase and the N-domain of the CDC48-like protein [175]. The best supported hypotheses of the disease mechanism of *VCP* mutations are disturbed ubiquitin–proteasome mediated protein degradation [31], autophagy [74], or both [75]. FTLD patients with a *VCP* mutation are associated with TDP-43 proteinopathy type D [176] characterized by large numbers of NCI, NII and DN in affected neocortical regions [99] (Fig. 3g–i). Some inclusions also stain for VCP protein p97 [140].

Symptoms of FTLD due to a *VCP* mutation become apparent in the mid-50s in 25–30 % of the IBMPFD patients [76, 78, 174]. The incomplete penetrance of the three clinical characteristics IBM, PDB and FTLD is independent of the underlying mutation [76, 78, 108, 174]. However, in FTLD, *VCP* mutations are rare and represent less than 1 % of the familial FTLD (Table 2). The most frequently reported FTLD subtypes are bvFTD and SD [176]. Interestingly, a single study reported *VCP* mutations in 1–2 % of the ALS patients in the absence of dementia symptoms [72, 77].

**Table 2:** Known FTLD genes

Gene symbol	Chromosomal location	Gene name	Mutation frequency (%)^a^
*C9orf72*	9p21.2	Chromosome 9 open reading frame 72	14–48
*GRN*	17q21.32	Progranulin	3–26
*MAPT*	17q21.32	Microtubule-associated protein tau	0–50
*CHMP2B*	3p11.2	Chromatin modifying protein 2B	<1
*VCP*	9p13.3	Valosin-containing protein	<1
Total			>17

Chromosomal localization, gene symbol and name and estimated mutation frequencies in familial FTLD patients

^a^Mutation frequency ranges were extracted from literature: *C9orf72* [101], *GRN* [52], *MAPT* [123]

### CHMP2B

Linkage analyses in a large Danish FTLD family identified a mutation in *CHMP2B* at chromosome 3p11.2 [147]. *CHMP2B* encodes a component of the heteromeric ESCRT-III complex with functions in the endosomal–lysosomal and the autophagic protein degradation pathway. The gene is expressed in neurons of all major brain regions. Mutations affect the C-terminal end of the protein due to aberrant splicing.

*CHMP2B* mutations were associated with enlarged vacuoles in cortical neurons in the frontal, temporal, parietal and occipital cortices, due to impaired endosome–lysosome fusion [160], and impairment of autophagy [25]. Ubiquitin-immunoreactive NCI do not stain for tau, TDP-43 or FUS antibodies [65, 66], consistent with a pathological classification of FTLD-UPS [100].

The general clinical diagnoses in patients of the large Danish family corresponded to bvFTD, with early personality changes being the most common feature [60]. In other patients, progressive aphasia involvement was described, although no diagnosis of PNFA, SD or LPA could be made. The aphasia is characterized by a reduction in spontaneous speech sometimes leading to mutism and preserved reading and repetition, most consistent with a dynamic aphasia [70, 167]. The average onset age is 58 years, ranging between 46 and 65 years [60, 70]. The *CHMP2B* p.Gln206His mutation was reported in two ALS patients [25, 117]. Other missense mutations have been described in patients with FTLD and/or MND; however, their pathogenic nature remains unclear [30].

### TARDBP and FUS

Mutations in the genes encoding the TDP-43 (*TARDBP*) and fused-in-sarcoma (*FUS*) proteins are typically associated with ALS. *TARDBP* mutations were initially identified [155] as a direct consequence of the identification of TDP-43-derived protein species as the major constituent of the aggregates found in upper and lower motor neurons of ALS patients without *SOD1* mutations and in FTLD-U [4, 117]. Whereas 5 % of the familial ALS patients have a *TARDBP* mutation, mutations are rarely found in FTLD and FTD-MND [8, 10].

TDP-43 is an RNA-binding protein that forms heterogeneous nuclear ribonucleoprotein complexes (hnRNP) which function in RNA processing activities of several cellular functions, including transcription, RNA splicing and microRNA processing [16, 17, 118]. Missense mutations were found in the C-terminal glycine-rich region involved in protein–protein interactions [118]. Similar to *TARDBP*, *FUS* is a member of the hnRNP family. Its location at chromosome 16p11.2, made it an excellent candidate gene to explain previously established genetic linkage to the same chromosomal region in multiple ALS families [137, 139] and mutation analyses of patients in these and other families identified *FUS* mutations [80]. Although *FUS* represents an ALS gene, a mutational analysis of the *FUS* gene in 122 patients with FTLD revealed one novel p.Met254Val mutation in a patient with pure bvFTD. The silico analysis of this missense mutation predicted a pathogenic affect; however, the biologic relevance of this mutation remains elusive [157, 171].

**Fig. 4:**
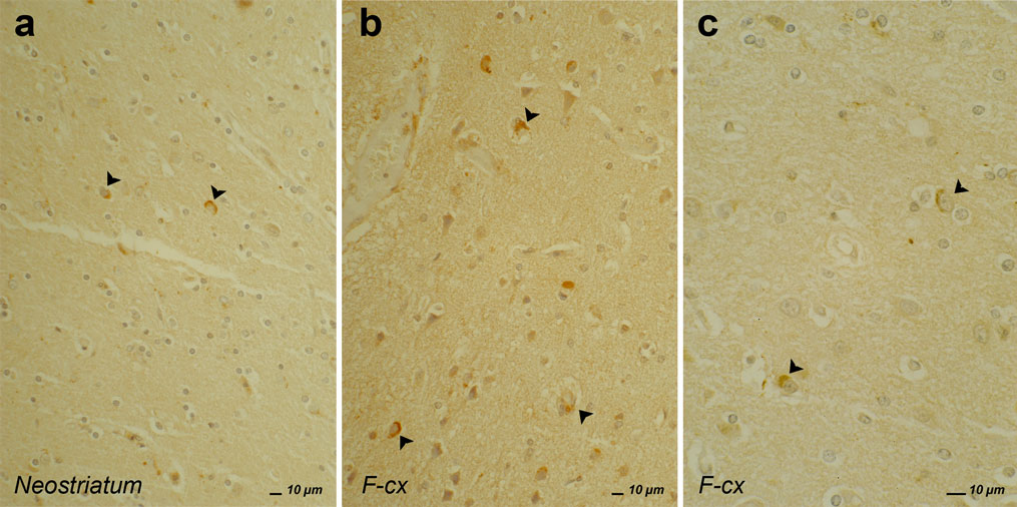
aFTLD-U patient with FUS pathology without any known mutation after immunostaining with FUS antibody. The *arrowhead* shows the kidney-shaped NCI in affected neostriatum (**a**) and frontal cortex (**b**, **c**, higher magnification)

Interestingly, an accumulation of FUS protein in inclusion bodies in neuronal cytoplasm and nucleus was associated with three clinicopathological subtypes of FTLD, defined by specific characteristics and location of NCI and NII [92, 97, 98, 114]. The first type is pathologically characterized by severe atrophy of the caudate nucleus and the frontotemporal cortex. Compact, round to oval kidney-shaped NCI and vermiform NII are localized in neocortex, granule cells of the dentate gyrus, striatum and to a lesser extent in globus pallidus, thalamus and periaqueductal gray matter (Fig. 4a–c). The cerebellar cortex is never affected. NCI and NII are not detected upon hematoxylin–eosin staining and were not immunoreactive to intermediate neurofilament on IHC. This type of FUS pathology is associated with a severe clinical syndrome resembling bvFTD and is referred to as atypical FTLD-U (aFTLD-U) [94]. Onset of disease is early, in 30s or 40s, and disease duration is approximately 7 years [85]. aFTLD-U is the most frequent FTLD-FUS subtype, accounting for 7–20 % of FTLD-U cases [94, 131, 142, 161]. The second FTLD-FUS type is characterized by an asymmetric atrophy of frontotemporal cortex and neostriatum, which is less severe than in aFTLD-U [85]. NCI and NII stain with FUS antibodies and to a lesser extent with antibodies against type IV interfilaments, alpha-internexin and neurofilaments. Therefore, this FTLD-FUS type is referred to as neuronal intermediate filament inclusion disease (NIFID) [94, 113]. Clinically NIFID patients usually develop a rapidly evolving FTD syndrome, mostly bvFTD, in combination with motor disorders such as parkinsonian or motor neuron symptoms. Patients usually develop the disease between 40 and 60 years of age, but earlier onset has been described and disease duration is on average approximately 3 years [85]. Considerable neuropathological similarities exist between aFTLD-U and NIFID; however, distinctive differences are observed. In NIFID, FUS-immunoreactive NCI are more extensive and widespread and are particularly numerous in CA1 and subiculum, but not in the granular layer of the dentate gyrus. The NCI morphology in NIFID patients is very heterogeneous, varying from small round to oval or tangle-like and annular shapes [73, 98, 100]. While less FUS-immunoreactive inclusions are seen in aFTLD-U, microscopic evaluation of aFTLD-U patients often shows sclerosis of the hippocampus and subcortical structures, for example, caudate nucleus, putamen and substantia nigra [85]. Another difference could be the presence of the NII, which are variably found in the hippocampus, but rare in other brain regions of NIFID patients, and are far more numerous in aFTLD-U patients [98, 100]. In the third FTLD-FUS type, referred to as basophilic inclusion body disease (BIBD), basophilic NCI staining with hematoxylin and eosin are seen in the pontine nuclei and to a lesser extent also in cerebral cortex [73, 98, 100, 106]. The basophilic NCI show FUS immunoreactivity in frontal cortex, basal ganglia and brain stem [98, 100]. BIBD presents with a clinical syndrome of early-onset ALS, occasionally accompanied with bvFTD.

## Susceptibility genes and risk loci

Compared with other complex neurodegenerative brain diseases including AD, little is known about susceptibility genes contributing to the risk of developing FTLD. This is mainly due to the fact that the familial component in FTLD is higher, and most research efforts aimed at the identification of Mendelian gene mutations. Also, the clinical and neuropathological heterogeneity of FTLD diseases have hampered large-scale genetic association studies in homogeneous patient series. One exception is the genome-wide association study in FTLD-TDP which has resulted in the identification of *TMEM106B* at chromosome 7p21 [165]. Nevertheless, other susceptibility genes are to be expected especially in the SD type of FTLD, in which family history is much less pronounced than in bvFTD and PNFA [32]. Consistent with this observation, mutations in the known FTLD genes were not associated with SD apart from a few atypical cases. Interestingly, SD is associated with a distinct type C of TDP-43 pathology suggesting a distinct disease mechanism [73]. FTLD-TDP type C is characterized by the presence of long DN localized in the superficial cortical layers, mainly layer II. NCI and NII are rare or absent compared to the other subtypes. Glial pathology is rare [99]. Together, these observations might suggest that SD is clinically, pathologically and genetically distinct from other types of FTLD and might predominantly be caused by the interaction of multiple yet unknown susceptibility genes.

*TMEM106B* was the only gene located in a single linkage disequilibrium block at 7p21 in which multiple single nucleotide polymorphisms (SNPs) were significantly associated in a series of 515 FTLD-TDP patients, a finding that was replicated in a second series of 89 FTLD-TDP patients [165]. In subsequent studies, association could be confirmed in one patient series [169], but not in another series [135]. It was found that *TMEM106B* may also contribute to the risk of developing FTLD in carriers of *GRN* mutations [165] possibly by modulating the levels of *GRN* secretion [26], however, other studies were not consistent with these findings [84, 169]. Interestingly, *TMEM106B* may also be associated with cognitive impairment in ALS [173].

TMEM106B is a type 2 integral membrane protein with unknown function, localizing to late endosomes and lysosomes [84]. The chromosome 7p21 risk haplotype was reported to act through altered *TMEM106B* gene expression in brain [165], although this was not confirmed in another study [169]. Interestingly, increased levels of TMEM106B were also associated with the inhibition of vacuolar H+-ATPases, a finding which may provide an unexpected biochemical link to GRN, since this protein is also strongly increased by the inhibition of vacuolar H+-ATPases [84].

Apart from the systematic genome-wide association study in FTLD-TDP [165], candidate gene association studies have reported genetic association of FTLD with other genes. In a series of pathologically confirmed FTLD-U patients without *GRN* mutations, a common genetic variant located in the 3′-untranslated region (UTR) of *GRN* in a binding site for miR-659 was identified as a major susceptibility factor for FTLD-U [126]. A significant reduction of GRN protein was observed in homozygous T-allele carriers in vivo, suggesting a similar mode of action as heterozygous loss-of-function mutations in *GRN* [126]. Another variant in the first intron of *GRN*, potentially affecting its expression, was also reported to be associated with FTLD in another patient series [49]. Nevertheless, other studies could not confirm the genetic risk for FTLD associated with variants in *GRN* [134, 152].

Besides harboring Mendelian FTLD mutations, *MAPT* was associated with risk of PSP [6, 64, 67], CBS [37, 67] and PD [40, 159], but inconsistent results were found in FTLD [9, 51, 86]. *MAPT* is represented in the human population as two genetically distinct haplotypes H1 and H2 due to its genomic location inside an inversion polymorphism [27]. The H1 haplotype is consistently overrepresented in 4R tau disorders, PSP and CBD [6, 67]. In young PSP patients, the risk was in part attributed to an SNP located in the large first intron of *MAPT*, which potentially modulated tau expression by modifying an LBP-1c/LSF/CP2 binding site. This transcription factor was shown to regulate the expression of genes in other neurodegenerative disorders [124]. A two-staged genome-wide association study in 2,165 PSP patients confirmed and extended these findings, and further implicated *STX6*, *EIF2AK3*, and *MOBP* in PSP [64]. Together, these genes suggested roles of vesicle-membrane fusion at the Golgi–endosomal interface, the endoplasmic reticulum unfolded protein response, and a myelin structure in PSP. The role of these genes in other tauopathies remains to be investigated.

## Summary

FTLD is a clinically, neuropathologically and genetically heterogeneous group of disorders with plenty of overlaps between the neurodegenerative mechanism and the clinical expression thereof. As many clinical syndromes belonging to FTLD tend to overlap especially in advanced disease stages, e.g. SD and bvFTD in the TDP-43 proteinopathies, aFTLD-U and NIFID in the FUS proteinopathies, identifying the symptomatology of the patient at an early stage of the disease course is crucial. In that respect, concerted evaluation of clinical and pathological parameters is helpful to obtain a precise diagnosis. The general clinical presentation of FTLD concurs with macroscopic characteristics of brain atrophy including localization, symmetry and degree of atrophy. The underlying neuropathological abnormalities, i.e. the proteinopathies are closely linked with the disease mechanism and are important diagnostic markers. Detailed information on the underlying neuropathology thus provides useful insights for early diagnosis, disease course prognosis and patient counseling and treatment that could not be obtained by clinical evaluation alone. Imaging, biological and genetic biomarkers are valuable parameters to assess the underlying pathological characteristics of the disease. Further research is needed to elicit additional biomarkers to extend the ability to characterize neuropathological causes of disease and to further specify clinicopathological correlations.

Despite immense progress in defining neuropathological abnormalities in FTLD diseases, not all FTLD proteinopathies are fully characterized yet. In the small group of FTLD-UPS patients, the protein accumulating in pathological aggregates remains to be identified. Similarly, not all mutations underlying the proteinopathies have been identified. Especially in the TDP-43 proteinopathies, several yet unknown Mendelian and susceptibility genes are expected to play a role. Specifically for FTLD-TDP type C, no gene has yet been identified. This type of proteinopathy is associated with clinical SD, in which family history was reported to be the least prominent and future genome-wide association studies may provide further insights in the genetics of this disease.
